# Supplementary material for: Reliability of the Linear Measurement (Contact) Method Compared with Stereophotogrammetry (Optical Scanning) for the Evaluation of Edema after Surgically Assisted Rapid Maxillary Expansion
Source: Healthcare (Basel). 2020 Mar 1;8(1):52. doi: 10.3390/healthcare8010052 (PMC7151041; doi:10.3390/healthcare8010052)
Supplement: Supplementary file 1 [file healthcare-08-00052-s001.pdf]

Supplementary Materials:

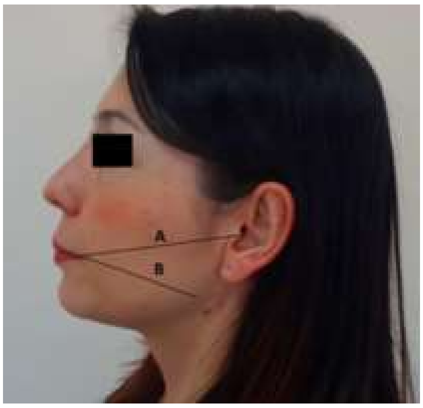

Figure S1. Distances between guide points A) Distance between the tragus and the corner of the mouth B) Distance between the angle of the mandible and the corner of the mouth.

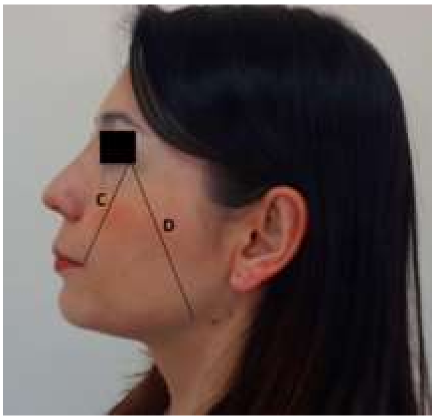

Figure S2. Distances between guide points C) Distance between the outer canthus of the eye and the corner of the mouth D) Distance between the outer canthus of the eye and the angle of the mandible.
